# The State Sanitary Conference

**Published:** 1907-11

**Authors:** 


A Monthly Review of Medicine and Surgery.

*EDITOR:*

WILLIAM WARREN POTTER, M. D.

All communications, whether of a literary or business nature, books for review and exchanges, should be addressed to the editor 238 DELAWARE AVE., BUFFALO, N. Y.

VOL. LXIII. NOVEMBER, 1907. NO. 4

### The State Sanitary Conference.

THE seventh annual conference of the Sanitary Officers of the State of New York held at Buffalo, October 16, 17, and 18, 1907, proved a most important meeting in the interests of preventive medicine. The object of these conferences, which are held yearly under the auspices of the State Department of Health, is to establish close relationship between the state and the local health authorities, to secure coöperation in the interests of sanitary science and to bring about a clearer understanding of the duties, powers and objects of the public health officers of the state.

The Buffalo meeting was a most satisfactory one, possibly the largest and most successful of any yet held. Nearly 600 health officers were present,—about one-half of the entire number in the state. The program was comprehensive and interesting. The first session, Wednesday afternoon, October 16, included an introductory address by Dr. Ernest Wende, health commissioner of Buffalo; addresses of welcome by Mayor J. N. Adam and President W. H. Gratwick, of the Buffalo Chamber of Commerce; an opening address by Dr. Eugene H. Porter, state commissioner of health; and finally, an address by Dr. Adna F. Weber, chief statistician of the state department of labor, who spoke in place of Governor Hughes, the duties of the latter detaining him at Albany.

Dr. Porter, speaking with reference to the consumption problem, recommended a thorough system of notification and registration, the establishing of district stations, state camps for consumptives and local hospitals for the chronic cases.

He also made the following reference to the Buffalo cancer research laboratory:

"It might be well, if we advised the citizens of the State not to put too much credence in the newspaper reports as to the curability or incurability of cancer. The laboratory here has been working faithfully and well on the problem of determining a probable cause for the disease. It has not concerned itself with attempting to find a cure. Its work, therefore, may not be popular, but it is scientific, and in the end will accomplish more than would a search first in this direction and then in that, for possible remedial agencies. When we have determined the cause of the malady, there is little doubt that a remedy will be forthcoming comparatively soon thereafter."

We shall print Dr. Porter's address in full in the December issue of the Journal.

Dr. Weber sketched the history of labor laws from their inception in England a century ago, dwelling particularly on the child labor law of New York State and the eight-hour law. Speaking on the former, he said examinations should be conducted with great care before certificates were issued, as on them depends the whole future of the child. He advocated the eight-hour law on the argument of health and expressed the hope that the state factory law, while as good as any in the United States, would be improved up to the point where it would be as valuable as that in force in Great Britain.

The evening's program, scheduled for 8 o'clock, was as follows:

The Dissemination and Control of Tuberculosis as Illustrated in the Bovine Species, Veranus A. Moore, M. D., professor of comparative pathology and bacteriology, New York State Veterinary College, Ithaca, N. Y. The Early Diagnosis and Treatment of Tuberculosis, John H. Pryor, M. D., Trustee New York State Hospital for Incipient Pulmonary Tuberculosis, Buffalo. The Social Aspect of Tuberculosis, Edward Devine, M. D., Secretary Charity Organisation Society, New York city.

Dr. Pryor's masterful paper contained much of general interest on the subject of pulmonary tuberculosis, from which we make the following excerpts:

"True effective prevention of tuberculosis is in its infancy; thus far it has been more or less a delusion. Little or nothing has been done in this state excepting in New York City. There great activity is displayed in a comprehensive campaign against the greatest scourge of humanity, and the results may afford the best test of the preventive measures to be found in the world. Ultimately the problem of tuberculosis will have to be solved by the health officers. They have the power and should assume the responsibility. Only organisation, effective plans and continuous, not spasmodic, activity will win.

We know that the disease can be detected early and that about 80 per cent. of those afflicted might recover if rational methods of relief were employed. The problem is colossal and must be attacked by broad methods. It does not involve a mere bacteriological question, but a sociological reformation. The slogan of battle,—sunlight and fresh air,—will be changed to food, sunlight and fresh air. Eighteen meals of oxygen a minute must be accompanied by three square meals of food. The poor cannot live by air and sunlight, and if the cost of existing, not living, continues to increase, consumption will be given greater opportunity to attack the underfed.

"The consumptive has a right to insist upon his chance. Why doesn't he demand it? Because he does not know, and generally is not told, that he is a consumptive until too late. Why does such an insignificant number receive an opportunity to recover and why is the disease not recognised in time? There is nothing to be gained by quibbling or evading the answer. A large proportion of the medical profession does not, or cannot, or will not, detect the presence of pulmonary tuberculosis during the incipient stage. There can be no other explanation of certain deplorable conditions, and the best remedy lies in honest admission. It has been claimed that many poor patients do not consult a physician at the right time, and this is sometimes quite true, but the comparative number is small. With very few exceptions the unfortunate patients who failed to gain admission to Ray Brook had been under the observation of one or more physicians for months, and most of them had been treated for bronchitis, catarrh and obstinate cold, or something else."

The program for Thursday, October 17, was a large one, and after doing full justice to the several topics the day ended with a banquet at the Ellicott Club, at which nearly 500 guests were seated. State Health Commissioner Porter acted as toastmaster; Dr. Ernest Wende read a poem and Senator Henry W. Hill, of Buffalo, spoke in place of Senator William J. Tully, of Corning, who was unavoidably detained at home.

In introducing Senator Hill, Commissioner Porter paid him the compliment of having done more than any member of the State Senate for public health.

Mr. Hill deplored the fact that New York State last year appropriated but $100,000 for the health of 8,000,000 people. He stated that Buffalo had one institution of which the state and the nation could well be proud, the laboratory for cancer study. Mr. Hill further stated that he had received letters from the greatest specialists in cancer diseases from every great university and all were unanimous in their expression that Buffalo led the world for scientific investigation of a disease that annually carries away 20,000 people.

Dr. Dewitt G. Wilcox, of Buffalo, made a speech abounding in clever wit and humor and the last speaker, Joseph W. Lawson,

Esq., of Albany, delivered a telling speech, which was notable for its oratorical finish and humorous allusions.

Friday's program included a visit to the stockyards, where the visitors witnessed the methods of inspecting, killing and caring for beeves at the Dold Packing House. While the banquet was being held on Thursday evening, Dr. Francis E. Fronczak, deputy health commissioner of Buffalo, spoke at convention hall to members of labor unions on the relation of working men to tuberculosis. At the session Friday evening, the audience listened to a symposium on pure milk and how to secure it. The addresses of special importance on this topic were delivered by Dr. Thomas Darlington, health commissioner of New York, and Dr. Ernest Wende, health commissioner of Buffalo.

During the entire progress of the conference, beginning even a day in advance of it, a tuberculosis exhibit was in continuous session at convention hall. Officers of the charity organisation society and others were in attendance describing the exhibit and explaining its purposes.

The opinion was expressed by those competent to judge that this conference was the most important of any yet held; certainly, it was of a character to reflect great credit upon Dr. Porter, the state commissioner of health, his assistants, and all who contributed in speech, address and in other ways to make the meeting so admirably fulfil its purposes.

---

THE Albany Sanitary Bureau embracing a group of sanitary experts, has been established for the purpose of making investigations, reports and recommendations relating to public and private water supply—quality, protection, purification; stream pollution, prevention and improvement; sewerage and sewage disposal; ventilation, heating, plumbing, fire protection, and the like, for public buildings; sanitary condition and improvement of dairies and creameries.

All communications should be addressed to the secretary, Olin H. Landreth, Professor of Engineering, Union College, Schenectady, N. Y.

---

HERE'S a new germ scare: A Copenhagen physician has discovered that the act of weeping, according to *The Tribune,* the releasing of tears that chase down the cheeks, moistens and sets in motion a host of harmful bacilli likely to injure those in the room with the weeping one. Especially does one who essays the role of comforter expose himself to danger from the liberated bacilli. "Is this latest germ scare to make it more difficult than it now is to find sympathisers?" queries a philosopher. "Will every one flee, for reasons of self-preservation, from the woman who is clearing her own atmosphere by having a good cry? And if there are sound scientific facts at the bottom of this physician's rather sensational announcement there must be bacilli unlimited in the audience room of a theater during one of those moments of which it is afterward said there was not a dry eye in the house."

---

Dr. J. N. McCormack, of Bowling Green, Ky., in conversation with Drs. Brayton, Potter and other physicians, as related in the *Indianapolis News,* told a little story of the late General Benjamin Harrison. "During the Civil War," said the doctor, "Colonel Harrison—for he was then colonel—was for a time in command at Bowling Green. Many soldiers were sick, and he appropriated the hotel of the place, the Mitchell House, for use as a hospital. Mr. Mitchell murmured somewhat, but had to give way to military necessity, Colonel Harrison assuring him that he should be paid for the use of the house, even though Harrison should have to pay out of his own pocket. The war ended and the years went on. Mr. Mitchell was well-to-do and presented no claim. Finally, when Harrison became President of the United States, Mr. Mitchell concluded to send his bill directly to the President. He did so, and President Harrison sent him his check for the money."

Sir Henry Alfred Pitman, who has just entered upon his hundredth year, claims the distinction, according to *The Sphere,* of being the oldest physician in the United Kingdom. Sir Henry took his B. A. at Cambridge in 1831 and his M. D. degree sixty-seven years ago, and for the last half century he has been connected with the medical staff of St. George's Hospital, of which institution he is still senior consulting physician. He is old enough to remember the rejoicings after Waterloo. Sir Henry was knighted in 1883.

